# Physical Activity during COVID-19 in German Adults: Analyses in the COVID-19 Snapshot Monitoring Study (COSMO)

**DOI:** 10.3390/ijerph18020507

**Published:** 2021-01-09

**Authors:** Theresa Maertl, Freia De Bock, Lena Huebl, Cornelia Oberhauser, Michaela Coenen, Caroline Jung-Sievers, on behalf of the COSMO Study Team

**Affiliations:** 1 Institute for Medical Information Processing, Biometry and Epidemiology–IBE, Chair of Public Health and Health Services Research, LMU Munich, Elisabeth-Winterhalter-Weg 6, 81377 Munich, Germany; theresa-maertl@gmx.de (T.M.); coberhauser@ibe.med.uni-muenchen.de (C.O.); coenen@ibe.med.uni-muenchen.de (M.C.); 2 Pettenkofer School of Public Health, Elisabeth-Winterhalter-Weg 6, 81377 Munich, Germany; 3 Federal Centre for Health Education, Maarweg 149-161, 50825 Cologne, Germany; Freia.DeBock@bzga.de; 4 Department of Tropical Medicine, Bernhard Nocht Institute for Tropical Medicine, University Medical Centre Hamburg-Eppendorf, 20251 Hamburg, Germany; l.huebl@uke.de; 5 I. Department of Medicine, University Medical Centre Hamburg-Eppendorf, 20251 Hamburg, Germany

**Keywords:** COVID-19, physical activity, exercise, lockdown, health behaviours, coping strategies, family

## Abstract

The novel coronavirus (COVID-19) and the resulting outbreak response measures in Germany and worldwide led to severe limitations in everyday life. This affected all sorts of daily activities and the possibility for physical activity (PA), which represents a major coping strategy against stress. The objective of this study was to analyse PA in German adults during a total lockdown phase including school closures in April 2020 in certain subgroups and in relation to other coping strategies. Data from the COVID-19 Snapshot Monitoring (COSMO) survey, an online cross-sectional study with 1034 participants between 18 and 74 years, were utilised (14/15 April 2020). In addition to descriptive analyses, the odds of performing PA according to the World Health Organization (WHO) recommendations for adults (at least 2.5 h/week of at least moderate intensity) were analysed by univariate and multivariate logistic regression analyses. In total, 440 (42.6%) participants fulfilled this criterion. Participants with children <6 years were less likely to meet the WHO recommendation (OR = 0.51; 95% CI: 0.33–0.78), while those with a higher level of education, good coping behaviour, regular alcohol consumption, and being satisfied with life were more likely to meet the WHO recommendation. In conclusion, PA intervention strategies tailored to specific vulnerable subgroups such as individuals with low educational background and parents with young children are needed in future pandemic response.

## 1. Introduction

In December 2019, the novel coronavirus (COVID-19) emerged in Wuhan, China, and since then, the infectious disease has spread throughout the world. On 27 January 2020, the first case of infection was detected in Germany [1]. On 12 March 2020, COVID-19 was declared as a global pandemic by the World Health Organization (WHO) with almost 125,000 reported cases worldwide and more than 20,000 confirmed cases and 1000 deaths in the European Region [2]. As a result, countries all over the world implemented measures to mitigate the spread of the virus.

In Germany, similar to other countries, these pre-emptive measures included the call to avoid contact with other people, to stay at home, to keep distance from other people of about 1.5 to 2 m, to work from home if possible, and to solely leave the house for necessary reasons such as to commute to work, for the doctor’s appointments, or to run errands. Furthermore, all schools including kindergartens as well as public places such as restaurants and other service companies such as fitness centres were closed (status: 22 March to 19 April) [3]. The implementation of these public health measures is expected to have influenced positive health behaviour (i.e., sleep, physical activity) and negative health behaviour (i.e., alcohol consumption, tobacco and drug use) [4]. 

At the same time, the level of stress has risen strongly [5]. Adults in all age groups have been experiencing a variety of concerns in connection with the COVID-19 pandemic: uncertainty about the spread of the novel coronavirus, separation and isolation from the social environment, fear of losing a loved one, negative economic consequences, and the loss of freedom [6]. Critical life events such as the current COVID-19 pandemic lead to a break in everyday life and are accompanied by limited controllability of life, which increases feelings of stress [7]. 

In order to deal with this unusual situation and feelings, people resort on the one hand to adaptive, positive coping strategies, such as exchange via phone or internet with friends and family, on the other hand to maladaptive coping strategies, such as increased sedentary behaviour, alcohol abuse, smoking, or a negative change in eating behaviour [8,9,10].

Physical activity (PA) is regarded as a healthy and adaptive coping strategy that can help to reduce mental health problems and anxiety levels [11,12,13]. WHO defines PA as any bodily movement produced by skeletal muscles that requires energy expenditure [14]. PA refers to all movement including during leisure time, to get to and from places, or as part of a person’s work [14]. Exercise is a subset of physical activity that is planned, structured, and has the goal to improve or maintain physical fitness [14]. Regular exercise is also associated with emotional resilience in stressful situations because of lower levels of cortisol and heart rates [15]. Moreover, the promotion of PA is especially important as physical inactivity and sedentary behaviour increases the risk of chronic diseases and the risk of higher morbidity from COVID-19 [16,17]. For that reason, the German Federal Government still supported PA at home or outside in the fresh air without company during the lockdown situation [3]. However, most likely, this coping strategy has not been available for all groups to the same extent during the pandemic in April 2020. For instance, the closure of childcare facilities such as kindergartens or schools turned into a major challenge for working mothers and fathers with young children [18]. Moreover, other subgroups may not have had the space, knowledge, or ability to exercise at home.

Therefore, the objectives of this study were to analyse PA in German adults during the COVID-19 pandemic and to discuss potential implications for following lockdown phases or future pandemics.

## 2. Materials and Methods

### 2.1. Study Sample

This study is based on data of the serial cross-sectional COVID-19 Snapshot Monitoring (COSMO) Germany study that is funded by the University of Erfurt, the Leibniz Institute for Psychology Information (ZPID), the Robert Koch Institute (RKI), and the Federal Centre for Health Education (BZgA) [19], and supported by the WHO Regional Office for Europe [20]. COSMO is an ongoing project that started on 6 March 2020, and collects data on a weekly basis using a 15 min online questionnaire to monitor the psychological situation of the German adult population during the COVID-19 situation. Each data collection is a non-probability quota sample, representative of the German adult population regarding age × gender and federal state according to the German census. Participants are recruited via an external study sample provider according to ISO 26362: 2009-compliant online panel (respondi.de, https://www.iso.org/standard/43521.html). All individuals between 18 and 74 years of age completing the survey are eligible for inclusion. Participants are admitted to the survey or screened out on the first page on the basis of the quotas. All participants provide informed consent before starting the survey. They take part voluntarily and receive remuneration. Ethical approval was obtained by the institutional review board at the University of Erfurt (#20200501). All procedures performed in the COSMO studies involving human participants were in accordance with the ethical standards of the University of Erfurt institutional research committee and with the 1964 Helsinki Declaration and its later amendments or comparable ethical standards. A large sample size of *n* = 1000 was chosen to detect small effects and increase the probability of congruence between the distribution of the demographics in the sample and the German population. Given a sensitivity power analysis for zero-order correlations (*p* = 0.05), a sample size of *n* = 1000 is sufficient to detect correlation coefficients of (at least) r = 0.08 with sufficient power of 0.8 in each survey. The details of the study, including design, eligibility criteria, sources and methods of recruitment, and ethical standards have been described in detail in the study protocol [19].

The analyses represented here derive from the 7th wave, which was collected on 14 and 15 April 2020 during the first total lockdown situation in Germany. This survey resulted in a dataset of responses from 1034 individuals (530 women, 504 men) and included information on participants’ demographics, PA, coping strategies, life satisfaction, self-efficacy, and perceived burden.

For comparison, reference data for PA from the cross-sectional German Health Update (GEDA) study 2014/2015, a national health survey of 24,016 adults aged 18 years and above with permanent residency in Germany, were used [21]. In the GEDA study, participants were randomly recruited from 301 communities in Germany, took part voluntarily, and completed the questionnaire on paper or online. The study took place from November 2014 to July 2015 [21].

### 2.2. Variables and Measures

#### 2.2.1. Demographics

Demographics, such as gender (i.e., male, female), age (i.e., 18–29, 30–44, 45–54, 55–64, ≥65 years), educational level (i.e., university entrance qualification/A-Level, no university entrance qualification/A-Level), children <6 years (i.e., yes, no), current relationship/marriage (i.e., yes, no), migration (i.e., yes, no, I don’t know), household language other than German (i.e., yes, no), household size (i.e., just me, 2 persons, ≥3 persons), and number of inhabitants (i.e., <20,000, 20,001–100,000, 100,001–500,000, ≥500,000) were assessed.

#### 2.2.2. Health-Related Covariates

Having a chronic disease and life satisfaction were considered as health-related covariates. Participants were asked if they had a chronic disease (i.e., yes, no, I don’t know) and their satisfaction of life was assessed on a 7 point Likert-type scale (1—completely dissatisfied, 7—completely satisfied). For the analysis, the 7 point Likert-type scale was recoded into groups of 1–3 (i.e., dissatisfied), 4 (i.e., neutral), and 5–7 (i.e., satisfied).

#### 2.2.3. Alcohol Consumption

Alcohol use was measured by asking participants about their regular alcohol consumption in the last 12 months and their alcohol consumption in the previous 4 weeks (during COVID-19). Participants were asked how many times a week they drank alcohol, such as beer, wine, sparkling wine, spirits, cocktails, alcoholic mixed drinks, liqueurs, or homemade alcohol (i.e., every day, several times per week, once a week, rarely, never).

#### 2.2.4. Coping Strategies

Coping skills and opportunities in connection to the currently limited contact possibilities were measured: “I make phone calls or exchange information with family, friends and acquaintances via digital media”, “I receive support offers from family, friends or neighbours”, ”I offer help to others, such as neighbourhood help with shopping”, “I have a plan for my daily routine in terms of sleep, work, or physical activities”, “I am bored”. Participants were asked to what extent these statements apply to their current situation. Answers were made on a 7 point Likert-type scale (1—strongly disagree, 7—strongly agree). Furthermore, the perception of COVID-19 was assessed: “The novel coronavirus is “1—something I feel helpless with, 7—something I can actively do something about”. For the analysis, the 7 point Likert-type scale was recoded into groups of 1–3 (i.e., no), 4 (i.e., neutral), and 5–7 (i.e., yes).

#### 2.2.5. Current Burden and Self-Efficacy

Participants were asked whether they experienced their personal situation as stressful at the moment (i.e., yes, no) and about their self-efficacy to avoid the coronavirus (1—very difficult, 7—very easy). For the analysis, the 7 point Likert-type scale was recoded into groups of 1–3 (i.e., difficult), 4 (i.e., neutral), and 5–7 (i.e., easy).

#### 2.2.6. Physical Activity (Outcome Variable)

The WHO recommends at least 2.5 h of moderate intensity aerobic PA throughout the week and muscle-strengthening activities on two or more days per week for adults between 18 and 64 [22]. In this study, PA was assessed using two items of the European Health Interview Survey-Physical Activity Questionnaire (EHIS-PAQ) that were also used for the GEDA study [21] and are based on the WHO recommendation. The EHIS-PAQ is a short, domain-specific PA questionnaire based on PA questions that have been used in large-scale health interview surveys before [23].

Moderate intensity aerobic PA was defined as sport or fitness in leisure time, which leads at least to a slight increase in respiratory or heart rate. It was measured by asking participants: “For the following question, think of sports, fitness, or physical activity that result in at least a slight increase in respiratory or heart rate—for example, (Nordic) walking, ball sports, jogging, cycling, swimming, aerobics, rowing, or badminton. How much time do you spend in total in a typical week in the current corona situation with sports, fitness, or physical activity in your free time?” (Hours, minutes per week; integer).

In addition, muscle-strengthening activities were assessed by asking, “On how many days in a typical week in the current corona situation do you perform physical activities specifically to build or strengthen muscles? For example, weight training or strengthening exercises (with weights, stretch bands, own body weight), knee bends, push-ups or sit-ups.” (Days per week; integer).

In this study, meeting the recommended 2.5 h of moderate intensity aerobic PA was chosen as the main outcome because of its association with the prevention of chronic diseases and the reduction of mental health problems.

Performing PA of moderate intensity for at least 2.5 h per week (WHO recommendation) was the main outcome variable. Performing muscle-strengthening activities at least two days a week (WHO recommendation) was considered as an additional secondary outcome.

### 2.3. Statistical Analysis

For descriptive statistics, absolute and relative frequencies for categorical variables and means and standard deviations (SD) for continuous variables were calculated. In addition, descriptive statistics were presented separately for participants with ≥2.5 or <2.5 h of moderate intensity aerobic PA per week.

For different groups, e.g., gender (e.g., male vs. female), the frequencies and proportions of persons with PA ≥ 2.5 h per week or muscle-strengthening activities ≥ 2 days per week together with its 95% confidence intervals (CI) were calculated. To investigate whether there are subgroups (e.g., male vs. female) that are more likely to meet the WHO recommendation, univariate logistic regression models and the resulting Odds Ratios (OR) including 95% CIs were computed. Furthermore, a univariate logistic regression analysis stratified by gender was performed for PA of moderate intensity.

In addition, data on moderate intensity aerobic PA and muscle-strengthening activity were compared in tabular form for age groups and gender with reference values from 2014/2015 from the GEDA study using proportions and 95% CIs. A difference was interpreted as statistically significant, where CIs did not overlap.

Three logistic regression models with multiple independent variables were conducted, and the resulting ORs including 95% CIs were presented. In Model 1, only socioeconomic variables (i.e., gender, age, highest education, relationship status, children <6 years) were included. In Model 2, health-related covariates (i.e., chronic disease, life satisfaction) were added in addition to the variables of Model 1. Model 3 presents the fully adjusted model, including active coping strategies (i.e., phone calls, offering help, having a plan for the daily routine) and alcohol consumption in addition to variables included in Model 1 and Model 2. Model fit was checked by using Pseudo R^2^ (Nagelkerke R^2^).

Statistical analyses were performed using SPSS 26.0 (IBM, Armonk, NY, USA) and the statistical program R [24], version 4.0.2 (Vienna, Austria). *p*-values of 0.05 or less were considered to be statistically significant.

## 3. Results

### 3.1. Sample Characteristics

A total of 1034 people completed the survey. Data on PA were complete for all cases. Table 1 lists participant characteristics in total and stratified based on the WHO recommendation of at least 2.5 h of PA per week. In total, 440 (42.6%) conducted at least 2.5 h of physical exercise per week. The mean age of the respondents was 45.8 (SD: 15.7) years. Of the participants, the majority was female (51.3%), in a relationship (69.4%), and had at least 10 years of education with university entrance qualification, A-Level (56.0%). Only 138 participants (13.3%) indicated that they had children <6 years. The majority of respondents had no migration background (86.7%), lived in a two-person household (42.7%), and in small towns with less than 20,000 inhabitants (37.9%). The average amount of PA of participants was 182.8 (SD: 285.4) min per week, and one-third (33.5%) of the population reported a chronic health condition.

### 3.2. Univariate Analysis: Absolute and Relative Frequencies and Odds Ratios

Table 2 shows absolute and relative frequencies (together with its 95% CI) and the corresponding odds ratios (including 95% CI) of those who were meeting the WHO recommendations by carrying out PA of moderate intensity for at least 2.5 h per week. Nearly half (42.6%, *n* = 440) of the study population met the WHO recommendation. The youngest showed the highest proportion (47.3%) of active people who met the recommended 2.5 h. Results of the univariate logistic regression indicate that the odds of meeting the WHO recommendation of 2.5 h PA throughout the week was significantly higher for those with higher education (OR = 1.76; 95% CI: 1.36–2.26), with high satisfaction of life (OR = 2.45; 95% CI: 1.72–3.50), high self-efficacy (OR = 1.45; 95% CI: 1.09–2.62), and good coping behaviour such as having phone calls (OR = 2.06; 95% CI: 1.35–3.16), offering help (OR = 1.85; 95% CI: 1.41–2.44), having a plan for daily routine (OR = 2.56; 95% CI: 1.84–3.53), and perceiving the coronavirus as something you can actively do something about (OR = 1.49; 95% CI: 1.12–1.97). Interestingly, participants who reported to drink alcohol several times (OR = 2.27; 95% CI: 1.55–3.33) or once a week (OR = 1.69; 95% CI: 1.09–2.62) also had a significantly higher odds of meeting the recommended 2.5 h of PA per week. Individuals with chronic diseases (OR = 0.74; 95% CI: 0.56–0.96) and participants with children <6 years (OR = 0.64; 95% CI: 0.44–0.93) were significantly less likely to be in the active group.

#### Univariate Analysis Stratified by Gender

In addition, an analysis stratified by gender was performed to reveal potential differences (data not shown). In total, 42.9% (*n* = 216) male and 42.3% (*n* = 224) female participants met the WHO recommendation for PA of moderate intensity. The results of the univariate analysis show that only females with children <6 years (OR = 0.54, 95% CI: 0.33–0.89) and women who received help (OR = 0.67, 95% CI: 0.46–0.99) had significantly lower odds of meeting the recommended 2.5 h of PA per week. Male participants between 30 and 44 years (OR = 0.58, 95% CI: 0.34–0.99) and 45 and 64 years (OR = 0.60, 95% CI: 0.37–0.99) had significantly lower odds of conducting 2.5 h PA of moderate intensity per week. Being in a relationship (OR = 1.70, 95% CI: 1.14–2.52) or using phone calls as a coping strategy (OR = 2.78, 95% CI: 1.56–4.94) was associated with a significantly higher OR to achieve the WHO recommendation for men.

### 3.3. Multivariate Logistic Regression

Results of the multivariate logistic regression on PA ≥ 2.5 h are presented in Table 3. In the fully adjusted Model 3, higher education (OR = 1.52; 95% CI: 1.15–2.02), having children <6 years (OR = 0.51; 95% CI: 0.33–0.78), and being satisfied (OR = 1.76; 95% CI: 1.19–2.60) or at least neutral (OR = 1.73; 95% CI: 1.08–2.75) with life remained significant. Among the added coping strategies, offering help to others (OR = 1.53; 95% CI: 1.13–2.06), having a plan for the daily routine (OR = 1.92; 95% CI: 1.35–2.73), and drinking alcohol several times per week (OR = 2.11; 95% CI: 1.40–3.18) was associated with a significantly higher odds of meeting the WHO recommendation of 2.5 h of moderate intensity PA. Gender, age, having a chronic disease, or using phone calls as a coping strategy were not significantly associated with PA at the multivariate level. The effects of the sociodemographic variables and health-related covariates in Model 1 and Model 2 have not changed considerably compared to the final Model 3.

### 3.4. Additional Analyses on Muscle-Strengthening Activities

To compare our analysis on PA of moderate intensity with muscle-strengthening activities, additional analyses were computed (data not shown). When taking muscle-strengthening activities at least twice a week as an outcome variable, 32.1% (*n* = 332; 95% CI: 29.3–35.0) of the participants met the WHO recommendation. This proportion is slightly but not significantly higher compared to reference values from the GEDA study, where 29.4% (95% CI: 28.6–30.2) of the participants met the recommendation for muscle-strengthening activities (Appendix A).

The results of the multivariate logistic regression models for muscle-strengthening activities remained comparable to those for PA of moderate intensity for the association with a higher level of education and good coping behaviours (data not shown).

## 4. Discussion

In this cross-sectional survey of 1034 German adults, we analysed PA behaviour during the lockdown situation as outbreak response to the COVID-19 pandemic. Overall, our findings show that only 42.6% of the population met the WHO recommendation of 2.5 h of PA per week in April 2020. Furthermore, our results suggest that participants with children <6 years and lower education were less likely to meet the WHO recommendation. Taken together, these were mainly groups with lower resources, either in terms of education, but also in terms of personal resources such as self-efficacy as well as social resources (e.g., getting help from others). In the specific lockdown situation with schools closed and grandparents and friends not available to help with childcare, parents of little children accumulate different lacking resources, among these primarily time to exercise and help in childcare. In contrast, a higher education, good coping behaviour, regular alcohol consumption, and high satisfaction of life were associated with significantly increased odds of meeting the recommended 2.5 h of PA per week. These results might help us understand whom to address to avoid a reduction of PA in further lockdown situations. In this study, we investigated what proportion and which population groups of German adults met the WHO recommendations on physical activity, focussing on health-promoting behaviors. People who do not meet this recommendation at all have to deal with the physical and psychological consequences of physical inactivity and this should not be ignored.

In addition, in other countries, studies have examined PA during the COVID-19 lockdown. In a recent study of 13,515 adults in Belgium, people who were classified as low active adults before reported exercising more during the lockdown and participants who were already highly active before COVID-19 reported exercising less during the lockdown [25]. During the so-called “lockdown light” in Belgium, schools, fitness, and health centres were closed, but people were still allowed and encouraged by the government to exercise at home or outside alone, with members of the same household, or with one friend [25]. In contrast, a cross-sectional study from Canada came to the conclusion that 40.5% of inactive participants became less active, whereas only 22.4% of active participants became less active during the lockdown in Canada [26]. The implemented public health measures in Canada were similar to those in Germany and included the closing of non-essential business services, national parks and playgrounds, and the request to practice social distancing and to stay home [26]. Moreover, a cross-sectional study from Spain investigated PA behaviour in 163 Spanish adults with chronic diseases who present an especially vulnerable group in the COVID-19 pandemic [27]. They found a significant decrease of moderate intensity PA in males and females with chronic conditions during the COVID-19 quarantine period, which forced the Spanish population to stay in their homes [27]. Thus, in most countries, PA levels decreased through lockdown, which was potentially associated with the implemented public health measures. This information is particularly crucial as higher levels of PA are associated with lower levels of anxiety and good mental health during COVID-19 [28,29].

Additional analyses were conducted to compare PA levels for age and gender with reference values from the GEDA study in Germany from 2014/2015 (Appendix A). Overall, 45.3% (95% CI: 44.2–46.4) of the participants of the GEDA study reported meeting the WHO recommendation for moderate intensity PA. This presents a slightly but not significantly higher proportion than in the COSMO sample of 14 and 15 April 2020, with 42.6% (95% CI: 39.5–45.6). Women between 45 and 64 years in the presented COSMO study showed the lowest proportion of active people with 37.2% (95% CI: 29.8–44.6). This percentage was significantly lower in comparison to the GEDA study, where women between 45 and 64 years showed with 47.8% (95% CI: 46.0–49.6) the highest proportion of participants who performed at least 2.5 h of PA. For other age groups or male participants, no significant difference was found. A report by the German Health Insurance (Deutsche Krankenversicherung, DKV) also examined the health behavior of Germans from 2010 to 2018 and found that physical activity that was measured in metabolic equivalent of task (MET) decreased during this time period [30]. Our comparison of COSMO data with representative data in Germany before the COVID-19 situation does not show a change of average PA levels. These results might be biased due to an overrepresentation of more highly educated people in our online sample. Nevertheless, governmental lockdown strategies might have to be differentiated to reduce contacts on the one side, but on the other side to assure PA possibilities outside for individuals.

Analysing the determinants of and possibilities to counteract the influence of lockdown measures on parents with young children might be also key for maintaining PA during lockdown. With the closure of kindergartens and no or only limited contact with grandparents or other childcare opportunities, the lives of parents, particularly of working mothers and fathers, has been severely affected. Thus, parents could only work from home or not at all during the lockdown, and they did not know how long this situation would last. This was also a difficult situation for children who no longer had access to playgrounds, sports clubs, or other group activities [18]. Adamo et al. [31] investigated the association of children of different ages on parental PA in 2315 Canadians in 2012 and came to a similar result to that of our study. The PA level of parents with young children (<6 years) was significantly lower than that of those without children, and these parents were less likely to meet the PA guideline of 150 min of moderate-to-vigorous PA per week [31]. However, the study of Adamo et al. was conducted at times without lockdown measures. Therefore, an already decreased PA behaviour in parents of young children might have been rendered more severe through the burdens of lockdown in the COVID-19 pandemic. In addition, our analysis stratified by gender showed that having a child <6 years of age was only significant for women, which suggests that the corona crisis also reveals and aggravates gender inequities. Not only are women still primarily responsible when it comes to childcare, elderly care and household chores [32]: the measures taken during lockdown also impede women to maintain PA levels, therefore fostering gender inequalities in the physical and mental health of mothers. Here, it is important to develop targeted programs to support these individuals. For instance, it would be conceivable to implement organised small neighbourhood-cohort sports programmes for parents together with their children in safe outdoor areas [25].

As expected, an overall good coping behaviour such as having phone calls, offering help, having a plan for daily routine, and perceiving the coronavirus as something you can actively do something about was also associated with a higher odds of meeting the PA recommendation of the WHO. A cross-sectional survey from Canada from 2014 that examined exercise as a coping behaviour for stress supports our results [33]. In this study, Canadians who reported using exercise for coping with stress were more likely to endorse other positive coping strategies and less likely to use alcohol or drugs for coping [33]. Moreover, in a cross-sectional study of 5545 Spanish adults during the current COVID-19 pandemic and lockdown, having a daily schedule was associated with lower levels of anxiety levels and depressive symptoms [34]. They came further to the result that a healthy diet, not reading about COVID-19 very often, taking the pursuing hobbies, and staying outdoors were effective coping behaviours and the best predictors of lower levels of depressive symptoms [34]. Apps and other digital tools that remind people to exercise might help to integrate PA into everyday life. Performing PA at home presents a good opportunity to avoid the coronavirus and maintain the one’s fitness level [29].

In addition, our results on the association of the level of education and PA are consistent with previous research. The GEDA study also found that individuals with a lower educational level were less likely to meet the WHO recommendation compared to individuals with a higher education level [21]. Therefore, health-promoting information, including digital tools, should be in plain language and accessible to the public in order to reach this target group as well [35]. Generally, approaches to keep up contact with population groups with low social, personal, and educational resources seem key in the pandemic. Intervention approaches forming small cohorts of peers within neighbourhoods, balancing out contact reduction needs with social needs important to lifestyle, physical, and mental health might be an option for next lockdown phases. Our study results found no difference between participants with or without an immigrant background and households in which German or another language is mainly spoken.

It is interesting to note that consuming alcohol several times per week was cross-sectionally associated with increased odds of meeting the WHO recommendation. The results of Cairney et al., where using exercise as a coping strategy was associated with less alcohol consumption, contradict these findings [33]. Other studies conducted before the corona crisis showed, similar to our investigation, a positive association between PA and alcohol consumption [36,37]. The results of our study may be explained by the fact that in times of social crisis such as the COVID-19 pandemic, the consumption of alcohol is another but negative coping mechanism used by people to calm stress and worries [8]. Additionally, the lack of social contacts and missing tasks could increase the consumption of alcohol. An anonymous online survey investigated the changes in alcohol drinking behaviour in 2102 German adults during the time of social restrictions [38]. Their findings suggest that 34.7% of the participants reported drinking more or much more alcohol since the beginning of the lockdown and especially low educated subjects and subjects with higher levels of perceived stress due to the lockdown are at risk of consuming more alcohol [38]. The rapid review of Brooks et al. [6] reported one study that assessed the effect of quarantine and social isolation on alcohol abuse or dependency symptoms. They came to the result that these factors were positively associated even 3 years after the SARS outbreak [39]. Thus, it seems necessary to inform the population about risks and possible long-term consequences of increased alcohol consumption and to establish social support services such as telephone or online counselling services. Moreover, it is vital that the health care system and social workers are aware of this problem and openly refer patients or clients to appropriate help services in case of an increase in alcohol consumption [40].

### Strengths and Limitations

One of the main strengths of our study is the large and well characterised quota sample that matches the German population in terms of age, gender, and residency. However, the COSMO study recruited from an online panel is not representative in terms of socioeconomic status of the population, with overrepresentation of well-educated groups. To the best of our knowledge, ours is the first study that reports PA behaviour in certain subgroups and in relation to further coping strategies in German adults during the COVID-19 pandemic.

However, some more potential limitations of this study should be considered. First, due to the cross-sectional study design, causal relationships cannot be drawn. Pre and post COVID-19 measures could be performed to see if the participants adapted their PA and coping behaviour because of the lockdown situation. The comparisons with the reference values from the GEDA study give an estimate of PA levels in times before the COVID-19 pandemic. However, the GEDA study, which was conducted from November 2014 to July 2015, measured PA over different seasons. Thus, the time effect must be taken into account in further research. Furthermore, the survey relies on self-reported data, which is vulnerable to recall bias and bias of social desirability. Especially for PA, under- and over-estimation could pose a problem in self-reported measures. Finally, although various covariates were included in our analyses, there may be other factors that are associated with PA behaviour during a lockdown situation.

## 5. Conclusions

In conclusion, the possibility and implementation of PA into daily life is differently distributed in different subgroups. Therefore, intervention strategies tailored to specific vulnerable subgroups such as adults with low educational background and families with young children should be in focus, as they were significantly less likely to be physically active during the lockdown situation. PA interventions could encompass focussing on safe neighbourhood areas and the provision of easily accessible health-promoting information and useful digital tools in plain language. Considering the health risks associated with physical inactivity, governments should leave untouched the right to leave home for physical activity outdoors during future lockdown situations.

## Figures and Tables

**Table 1:** Sample characteristics.

Characteristics	Total *n* = 1034		PA < 2.5 h per Week *n* = 594		PA ≥ 2.5 h per Week *n* = 440	
	*N*	%/Mean (SD)	*n*	%/Mean (SD)	*n*	%/Mean (SD)
Gender						
Male	504	48.7	288	48.5	216	49.1
Female	530	51.3	306	51.5	224	50.9
Age	1034	45.8 (15.7)	594	46.4 (15.5)	440	45.0 (16.0)
Age Category						
18–29	207	20.0	109	18.4	98	22.3
30–44	297	28.7	167	28.1	130	29.5
45–64	351	33.9	215	36.2	136	30.9
≥65	179	17.3	103	17.3	76	17.3
Highest Education						
A-Level	579	56.0	296	49.8	281	63.9
No A-Level	455	44.0	298	50.2	159	36.1
Relationship Status						
Yes	718	69.4	404	68	314	71.4
No	316	30.6	190	32	126	28.6
Children <6 years						
Yes	138	13.3	92	15.5	46	10.5
No	896	86.7	502	84.5	394	89.5
Migration ^a^						
Yes	133	12.9	76	12.8	57	13.0
No	896	86.7	515	86.7	381	86.6
Household Language						
German	795	76.9	454	76.4	341	77.5
Other than German	239	23.1	140	23.6	99	22.5
Household Size						
Just me	236	22.8	146	24.6	90	20.5
2 persons	442	42.7	243	40.9	199	45.2
≥3 persons	356	34.4	205	34.5	151	34.3
Inhabitants						
<20,000	392	37.9	227	38.2	165	37.5
20,001–100,000	257	24.9	148	24.9	109	24.8
100,001–500,000	181	17.5	103	17.3	78	17.7
≥500,000	204	19.7	116	19.5	88	20.0
Physical Activity						
Minutes per week	1034	182.8 (285.4)	594	32.6 (43.2)	440	385.6 (342.5)
Chronic Disease						
Yes	346	33.5	214	36.0	132	30.0
No	642	62.1	349	58.8	293	66.6
I don’t know	46	4.4	31	5.2	15	3.4

^a^: Five participants who indicated “I don’t know” as an answer are not presented in the analysis. Abbreviations: PA: physical activity, *N*: number of cases in the total sample, *n*: number of cases in the subsamples with different levels of PA, SD: standard deviation.

**Table 2:** Univariate analysis: absolute and relative frequencies and Odds Ratios—PA ≥ 2.5 h per week.

Characteristics	PA ≥ 2.5 h per Week (*n* = 440)				
	*n*	% of Subgroup	95% CI	OR	95% CI
Gender					
Female (reference)	224	42.3	[38.1–46.5]		
Male	216	42.9	[38.5–47.2]	1.03	[0.80–1.31]
Age Category					
18–29 (reference)	98	47.3	[40.5–54.1]		
30–44	130	43.8	[38.1–49.4]	0.87	[0.61–1.24]
45–64	136	38.7	[33.7–43.8]	0.70	[0.50–0.996] *
≥65	76	42.5	[35.2–49.7]	0.82	[0.55–1.23]
Highest Education					
No A-Level (reference)	159	34.9	[30.6–39.3]		
A-Level	281	48.5	[44.5–52.6]	1.76	[1.36–2.26] ***
Relationship Status					
No (reference)	126	39.9	[34.5–45.3]		
Yes	314	43.7	[40.1–47.4]	1.17	[0.90–1.53]
Children <6 years					
No (reference)	394	44.0	[40.7–47.2]		
Yes	46	33.3	[25.5–41.2]	0.64	[0.44–0.93] *
Migration					
No (reference)	381	42.5	[39.3–45.8]		
Yes	57	42.9	[34.5–51.3]	1.01	[0.70–1.47]
Household Language					
German (reference)	341	42.9	[39.5–46.3]		
Other than German	99	41.4	[35.2–47.7]	0.94	[0.70–1.26]
Household Size					
Just me (reference)	90	38.1	[31.9–44.3]		
2 persons	199	45.0	[40.4–49.7]	1.32	[0.96–1.83]
≥3 persons	151	42.4	[37.3–47.6]	1.20	[0.84–1.67]
Inhabitants					
≥500,000 (reference)	88	43.1	[36.3–49.9]		
100,001–500,000	78	43.1	[35.9–50.3]	1.00	[0.67–1.50]
20,001–100,000	109	42.4	[36.4–48.5]	0.97	[0.67–1.41]
<20,000	165	42.1	[37.2–47.0]	0.96	[0.68–1.35]
Chronic Disease					
No (reference)	293	45.6	[41.8–49.5]		
I don’t know	15	32.6	[19.1–46.2]	0.58	[0.31–1.09]
Yes	132	38.2	[32.8–43.0]	0.74	[0.56–0.96] *
Life Satisfaction					
Dissatisfied (reference)	51	26.7	[20.4–33.0]		
Neutral	76	42.2	[36.1–50.6]	2.01	[1.30–3.10] **
Satisfied	313	47.2	[43.4–51.0]	2.45	[1.72–3.50] ***
Phone Calls					
No (reference)	33	28.4	[20.2–36.7]		
Neutral	55	40.1	[31.9–48.4]	1.69	[0.99–2.86]
Yes	352	45.1	[41.6–48.6]	2.06	[1.35–3.16] ***
Receive Help					
No (reference)	225	42.9	[38.7–47.2]		
Neutral	75	44.6	[37.1–52.2]	1.07	[0.76–1.52]
Yes	140	40.9	[35.7–46.2]	0.92	[0.70–1.21]
Offer Help					
No (reference)	173	35.5	[31.2–39.7]		
Neutral	78	45.6	[38.2–53.4]	1.53	[1.07–2.18]
Yes	189	50.4	[45.3–55.5]	1.85	[1.41–2.44] ***
Plan for Daily Routine					
No (reference)	65	27.7	[21.9–33.4]		
Neutral	63	37.7	[30.4–45.1]	1.58	[1.04–2.42] *
Yes	312	49.4	[45.5–53.3]	2.56	[1.84–3.53] ***
Being Bored					
Yes (reference)	111	41.4	[35.5–47.3]		
Neutral	39	33.6	[25.0–42.2]	0.63	[0.42–0.95] *
No	290	44.6	[40.8–48.4]	0.88	[0.66–1.17]
The virus is something…					
…I feel helpless with (reference)	163	39.6	[34.8–44.3]		
Neutral	93	37.3	[31.3–43.4]	0.91	[0.66–1.26]
…I can actively do something about	184	49.3	[44.3–54.4]	1.49	[1.12–1.97] **
Alcohol Consumption					
Never (reference)	59	33.5	[26.6–40.5]		
Rarely	115	36.1	[30.8–41.6]	1.12	[0.76–1.65]
Once a week	76	46.1	[38.5–53.7]	1.69	[1.09–2.62] *
Several times per week	167	53.4	[47.8–58.9]	2.27	[1.55–3.33] ***
Every day	23	37.7	[25.5–49.9]	1.20	[0.66–2.20]
Experience Life Stressful					
No (reference)	265	42.8	[38.9–46.7]		
Yes	175	42.2	[37.4–46.9]	0.97	[0.76–1.25]
Self-efficacy: Avoiding the virus is					
Difficult (reference)	63	36.8	[29.6–44.1]		
Neutral	114	37.1	[31.7–42.5]	1.01	[0.69–1.49]
Easy	263	47.3	[43.2–51.5]	1.54	[1.08–2.19] *

* *p* < 0.05; ** *p* < 0.01; *** *p* < 0.001; marked in bold. Abbreviations: CI: confidence interval, PA: physical activity, *n*: number, OR: odds ratio, SD: standard deviation.

**Table 3:** Multivariate logistic regression models—PA ≥ 2.5 h per week.

	PA ≥ 2.5 h per Week (*n* = 440)					
Characteristics	Model 1		Model 2		Model 3	
	OR	[95% CI]	OR	[95% CI]	OR	[95% CI]
Gender						
Female (reference)						
Male	1.0	[0.78–1.29]	1.02	[0.79–1.32]	0.97	[0.73–1.27]
Age Category						
18–29 (reference)						
30–44	0.95	[0.66–1.38]	0.98	[0.67–1.42]	0.88	[0.59–1.30]
45–64	0.75	[0.52–1.08]	0.78	[0.54–1.14]	0.68	[0.46–1.00]
≥65	0.94	[0.61–1.44]	0.96	[0.62–1.51]	0.85	[0.73–1.27]
Highest Education						
No A-Level (reference)						
A-Level	1.73	[1.32–2.29] ***	1.66	[1.26–2.18] ***	1.52	[1.15–2.02] **
Relationship Status						
No (reference)						
Yes	1.27	[0.96–1.68]	1.08	[0.81–1.45]	0.93	[0.69–1.26]
Children < 6 years						
No (reference)						
Yes	0.53	[0.36–0.80] **	0.55	[0.36–0.82] **	0.51	[0.33–0.78] **
Chronic Disease						
No (reference)						
I don’t know			0.67	[0.45–1.30]	0.68	[0.35–1.35]
Yes			0.86	[0.65–1.15]	0.89	[0.66–1.20]
Life Satisfaction						
Dissatisfied (reference)						
Neutral			1.91	[1.22–2.98] **	1.73	[1.08–2.75] *
Satisfied			2.24	[1.54–3.24] ***	1.76	[1.19–2.60] **
Phone Calls						
No (reference)						
Neutral					1.19	[0.67–2.12]
Yes					1.35	[0.85–2.16]
Offer Help						
No (reference)						
Neutral					1.47	[1.00–2.16]
Yes					1.53	[1.13–2.06] **
Plan for Daily Routine						
No (reference)						
Neutral					1.23	[0.78–1.95]
Yes					1.92	[1.35–2.73] ***
Alcohol Consumption						
Never (reference)						
Rarely					1.01	[0.68–1.52]
Once a week					1.46	[0.92–2.33]
Several times per week					2.11	[1.40–3.18] ***
Every day					1.26	[0.66–2.40]
Pseudo R^2^	0.041		0.070		0.140	

To view the *n* of the subgroups refer to Table 2. * *p* < 0.05; ** *p* < 0.01; *** *p* < 0.001; marked in bold. Model 1: demographics (gender, age, education, relationship status, children <6 years). Model 2: + health-related covariates (chronic disease, life satisfaction). Model 3: + active coping strategies (phone calls, offer help, plan for daily routine, alcohol consumption). Abbreviations: CI: confidence interval, PA: physical activity, OR: odds ratio.

## Data Availability

Data are not publicly available but interested parties may contact the authors for more information. The data are not publicly available due to ethical restrictions.

## Appendix A. Comparison with Reference Values from the German Health Update (GEDA)

**Table A1:** Comparison of physical activity with reference values from the German Health Update (GEDA).

	Moderate Intensity PA ≥ 2.5 h per Week				Muscle-Strengthening Activities ≥ 2 Times a Week			
	2014/2015 ^a^		during COVID-19 (April 2020)		2014/2015 ^a^		during COVID-19 (April 2020)	
	%	95% CI	%	95% CI	%	95% CI	%	95% CI
Women (total)	42.6	[41.3–43.9]	42.3	[38.1–46.5]	27.6	[26.7–28.6]	30.4	[26.5–34.3]
18–29	45.2	[42.3–48.2]	42.7	[33.5–52.0]	34.5	[32.1–37.0]	45.5	[36.2–54.8]
30–44	38.8	[36.7–41.0]	47.1	[39.6–54.6]	21.1	**[19.5–22.9]**	30.8	**[23.9–37.7]**
45–64	47.8	**[46.0–49.6]**	37.2	**[29.8–44.6]**	29.4	**[27.9–30.9]**	21.3	**[15.1–27.6]**
≥65	36.5	[34.0–39.1]	41.7	[31.1–52.2]	26.4	[24.4–28.4]	27.4	[17.9–36.9]
Men (total)	48.0	[46.6–49.4]	42.9	[38.5–47.2]	31.2	[30.2–32.3]	33.9	[29.8–38.1]
18–29	56.7	[53.6–59.8]	52.6	[42.6–62.5]	43.9	[41.1–46.8]	49.5	[39.5–59.4]
30–44	44.8	[42.1–47.5]	39.2	[30.6–47.8]	28.5	**[26.2–30.8]**	43.2	**[34.5–51.9]**
45–64	45.6	[43.7–47.6]	40.1	[33.1–47.1]	26.3	[24.7–27.9]	26.2	[19.9–32.5]
≥65	48.3	[45.9–50.7]	43.2	[33.2–53.1]	32.2	**[30.2–34.4]**	21.1	**[12.9–29.3]**
Total (Women and Men)	45.3	[44.2–46.4]	42.6	[39.5–45.6]	29.4	[28.6–30.2]	32.1	[29.3–35.0]

^a^: Data from the GEDA study [21]. A difference is interpreted as statistically significant where confidence intervals do not overlap; marked in bold. Abbreviations: CI: confidence interval, PA: physical activity.
